# Effects of a Low Level Laser on Periodontal Tissue in Hypofunctional Teeth

**DOI:** 10.1371/journal.pone.0100066

**Published:** 2014-06-13

**Authors:** Hidetaka Hayashi, Akiko Terao, Ryo Kunimatsu, Toshitsugu Kawata

**Affiliations:** 1 Hiroshima Shuccho Dental clinic, Hiroshima, Japan; 2 Postgraduate Student, Department of Orthodontics and Craniofacial Developmental Biology, Hiroshima University Graduate School of Biomedical Science, Hiroshima, Japan; 3 Assistant Professor, Department of Orthodontics and Craniofacial Developmental Biology, Hiroshima University Graduate School of Biomedical Science, Hiroshima, Japan; 4 Char and chief professor, Department of Orthodontics, Kanagawa Dental University, Kanagawa, Japan; University of Palermo, Italy

## Abstract

Malocclusions, such as an open bite and high canines, are often encountered in orthodontic practice. Teeth without occlusal stimuli are known as hypofunctional teeth, and numerous atrophic changes have been reported in the periodontal tissue, including reductions in blood vessels in the periodontal ligament (PDL), heavy root resorption, and reduced bone mineral density (BMD) in the alveolar bone. Low Level Laser (LLL) has been shown to have a positive effect on bone formation and the vasculature. Although the recovery of hypofunctional teeth remains unclear, LLL is expected to have a positive influence on periodontal tissue in occlusal hypofunction. The aim of the present study was to elucidate the relationship between LLL and periodontal tissue in occlusal hypofunction. Twenty-four male rats aged 5 weeks were randomly divided into control and hypofunctional groups. An anterior metal cap and bite plate were attached to the maxillary and mandibular incisors in the hypofunctional group to simulate occlusal hypofunction in the molars. LLL irradiation was applied to the maxillary first molar through the gingival sulcus in half of the rats. Rats were divided into four groups; control, control+LLL, hypofunctional, and hypofunctional+LLL. Exposure to LLL irradiation was performed for 3 minutes every other day for 2 weeks. Animals were examined by Micro-CT at 5 and 7 weeks and were subsequently sacrificed. Heads were resected and examined histologically and immunohistologically. The hypofunctional group had obvious stricture of the PDL. However, no significant differences were observed in the PDL and alveolar bone between the hypofunctional+LLL and the control groups. In addition, the expression of basic fibroblast growth factor (bFGF) and vascular endothelial growth factor (VEGF)-positive cells were higher in the hypofunctional + LLL group than in the hypofunctional group. These results indicated that LLL enhanced the production of bFGF and VEGF in the periodontal tissue of hypofunctional teeth.

## Introduction

Malocclusions, such as an open bite, high canines, or under-occluded teeth, are often encountered in orthodontic practice. Teeth without occlusal stimuli are known as hypofunctional teeth, and numerous atrophic changes have been reported in the periodontal ligament (PDL) of these teeth [1], [2]. The extent of root resorption was also shown to be significantly greater in hypofunctional teeth than in control teeth under normal occlusal conditions during orthodontic tooth movement in rats [3]. Moreover, root size and the structure of the PDL may be reduced because of disuse atrophy resulting from defects in occlusal function [4]. The effects of the loss of occlusal stimuli loss on alveolar bone formation have also been reported [5].

Low Level Laser (LLL) has recently been shown to have a positive influence on diseases of the joints, connective tissue, neuronal tissue, bone formation, and vasculature [6], [7]. LLL was previously used to enhance bone healing after fractures [8], [9], and to stimulate condylar growth [10]. Moreover, LLL may accelerate the process of fracture repair or increase bone mineral density (BMD) [11]. These findings suggest that low level laser therapy (LLLT) may have a positive effect on newly formed bone. The use of low-level diode lasers in periodontal therapy has recently been considered to improve wound healing in gingival tissue and accelerates gingival healing after LLLT in sites undergoing gingivectomy [12], [13]. Due to their wavelength characteristics, low-level diode lasers are able to reach not only epithelial tissues, but also subepithelial tissues, and laser irradiation is also expected to induce the proliferation of osteoblasts [14] and periodontal ligament fibroblasts [15].

Numerous studies have reported structural changes in the PDL under hypofunctional conditions, and an association with cytokine growth factors that may affect the biological properties of hypofunctional teeth. Occlusal stimuli have been shown to regulate interleukin-1 beta and basic fibroblast growth factor (bFGF) [16]. On the other hand, recent studies have indicated that LLL irradiation is able to stimulate the release of growth factors, such as vascular endothelial growth factor (VEGF) [17], bFGF, and insulin-like growth factor-1 [18]. bFGF is known to promote the proliferation of various cells associated with wound healing, and plays important roles in the differentiation of mesenchymal cells into fibroblasts and osteoblasts, angiogenesis, and formation of the extracellular matrix [19], [20]. VEGF is the primary mediator of angiogenesis [21] and has various biological functions, such as increasing vascular permeability [22]. It has also been shown to be involved in bone remodeling [23], [24]. Therefore, bFGF and VEGF may play important roles in maintaining homeostasis in periodontal tissue. Thus, we focused on bFGF and VEGF when we evaluated the condition of the PDL in hypofunctional teeth.

The aim of the present study was to elucidate the relationship between LLL and periodontal tissue in hypofunctional teeth, and clarify the participation of bFGF and VEGF.

## Materials and Methods

### Ethics Statement

All experiments were approved by the Animal Experimentation Committee at Hiroshima University, and conformed to the ARRIVE guidelines for animal research [25] and Rules for Animal Experiments of Hiroshima University.

### Animals

**Figure 1:**
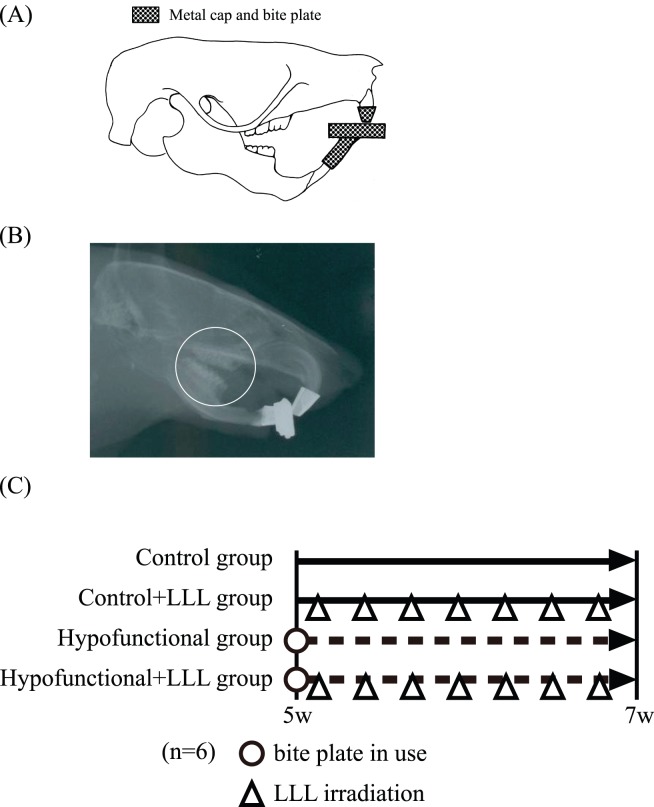
Experimental model and itinerary. (A) In order to eliminate occlusal force at the molar region, an anterior bite plate and metal cap were attached to the mandibular and maxillary incisors, respectively. (B) X-ray images were obtained to confirm the hypofunctional condition (○). (C) Itinerary.

Twenty-four 5-week-old male Wistar rats (Charles River Labs, Yokohama, Japan) were used. Rats were randomly divided into hypofunctional and control groups. In the hypofunctional group, an appliance consisting of a metal cap made of band material (3M Unitek Co., Tokyo, Japan) and an anterior bite plate made of a new ST lock base (Dentsply-Sankin, Tokyo, Japan) were bonded with composite resin (Clearfil Majesty LV; Kuraray Co., Ltd., Kurashiki, Japan) onto the maxillary and mandibular incisors, respectively [4] (Fig. 1A). The appliance was used for 2 weeks in the hypofunctional group. LLL irradiation was applied to the maxillary first molar through the gingival sulcus in half of the rats (Lumix2™ HEPL, Fisioline s.r.l.; Verduno, Cuneo, Italy) (48.6 J; frequency: 30 kHz). Rats were then divided into four groups; control, control+LLL, hypofunctional, and hypofunctional+LLL. Exposure was performed for 3 minutes every other day for 2 weeks. Animals were subjected to Micro-CT (SkyScan1176; SkyScan, Kontich, Belgium) at 5 and 7 weeks, and were then scarified. Rats in the hypofunctional group were subjected to soft X-ray radiography to confirm occlusal conditions at age 5 and 7 weeks (Fig. 1B). Next the heads were then resected and examined histologically and immunohistologically. The experimental itinerary is summarized in Figure 1C.

All animals were fed a powder diet (Rodent Diet CE-2; Japan CLEA Inc., Tokyo, Japan), and water *ad libitum* under a 12-hour light/dark environment at a constant temperature of 23°C. Rats were weighed once a week during the experimental period.

### Laser Device and Irradiation

We used a low-level diode laser (Lumix2™ HEPL, Fisioline s.r.l.) that emits pulse waves at a wavelength of 904–910 nm with a peak power of 45 W, maximum pulse repetition rate of 30 kHz, and pulse duration of 200 ns.

LLL was applied with a probe (diameter, 8.0 mm) possessing a free program mode around the maxillary first molar through the gingival sulcus in the control+LLL and hypofunctional+LLL groups (48.6 J; frequency: 30 kHz). Exposure was performed for 3 minutes every other day for 2 weeks.

### Morphological Analysis of PDL Thickness and BMD of the Maxillary Alveolar Bone by Micro-CT

**Figure 2:**
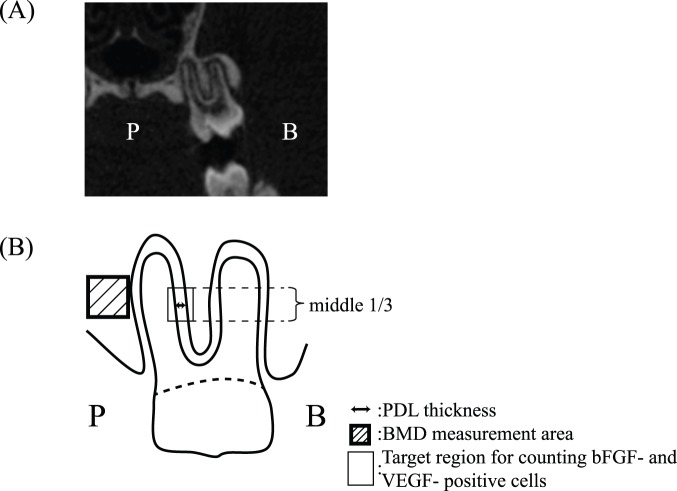
Measurement items in detail. (A) Micro-CT images of rat periodontal tissue. (B) Measurement items in detail. PDL thickness of the distal palatal root. BMD in the alveolar bone on the palatal side (500 µm^3^). Counting bFGF- and VEGF-positive cells. The middle 1/3 of the buccal aspect of the distal palatal root was selected for observations. A rectangular area (300×400 µm) including PDL cells and alveolar bone lining cells was used for measurements. B: buccal, P: palatal.

The thickness of the PDL at the distal palatal root of the maxillary first molars was measured using of Micro-CT at 5 and 7 weeks of age. Image reconstruction on an appropriate cross-section was performed using software (Nrecon; SkyScan), and the thickness of the PDL on the buccal side of the distal palatal root of the maxillary first molars was measured (Data Viewer; SkyScan) (Fig. 2A). The BMD of the maxillary alveolar bone on the palatal side was analyzed (500 µm^3^) (CT-An; SkyScan), and measurement items are summarized in Figure 2B. The same researcher performed all measurements. Measurements were repeated 3 times, and mean values were used.

### Tissue Preparation

Animals were deeply anesthetized in diethyl ether, followed by an intraperitoneal injection of chloral hydrate (400 mg/kg), and were then were subjected to soft X-ray radiography to confirm the occlusal condition prior to being sacrificed by means of transcardiac perfusion with 4% paraformaldehyde. Maxillae were immediately immersed in the same fixative solution overnight at 4°C. Tissue blocks were subsequently decalcified in 14% ethlylene diamine tetraacetic acid (EDTA) at 4°C for 4–6 weeks and prepared for the paraffin-embedded method. Serial sections of 5.0 µm in thickness were prepared along the frontal fault, perpendicular to the long axis of the distal root of the maxillary first molar. Sections were prepared for hematoxylin-eosin (H-E) and immunohistochemical staining.

### Immunohistochemical Staining

After deparaffinization, sections from 7-week-old rats that included the root canal were treated with 3% hydrogen peroxide in absolute methanol to block endogenous peroxidase. Sections were immunostained with a 1∶50 dilution of primary anti-rat bFGF rabbit polyclonal antibodies (Santa Cruz Biotechnology, Inc., CA), followed by the anti-rabbit secondary IgG antibody (Hystofine simple stain rat MAX-PO(R); Nichirei, Tokyo, Japan), and were immunostained with a 1∶150 dilution of primary anti-rat VEGF chicken polyclonal antibodies (Abcam, Inc., CA), followed by an anti-chicken secondary IgG antibody (Abcam). Immunoreactive sites were finally visualized with 3, 3′-diaminobenzidine (DAB). Counterstaining was performed using hematoxylin. Sections incubated without the primary antibody were used as a negative control.

### Number of bFGF- and VEGF-immunopositive PDL Cells

The middle one-third buccal aspect of the PDL on the distal palatal root was photographed using an optical microscope (Biozero; Keyence). Quantitative images were measured using image analysis software (BZ analyzer; Keyence). The number of bFGF- and VEGF-positive PDL cells was counted in a rectangular area (300×400 µm) (Fig. 2B). Three representative sections from each of the five samples of all groups were measured in a blinded manner.

### Statistical Analysis

PDL thickness, BMD of the maxillary alveolar bone, and number of bFGF- and VEGF-immunopositive PDL cells were measured in each group, and characteristic values in the experimental groups were compared with those in the control groups. To determine the significance of differences among groups of rats, we performed a repeated one-way analysis of variance (ANOVA) and the Tukey-Kramer test using a Statview Confidence level of *p*<0.05.

## Results

### Body Weight

All animals exhibited normal growth, and no significant difference was observed among the experimental groups (data not shown).

### Occlusal Condition

In order to confirm occlusal contact in the molar region, soft X-ray images were taken at 5 and 7 weeks of age before sacrifice in each group (Fig. 1B). Occlusal hypofunction was confirmed in the molars of all rats in the hypofunctional and hypofunctional+LLL groups.

### Morphometric Findings from Micro-CT Analyses

**Figure 3:**
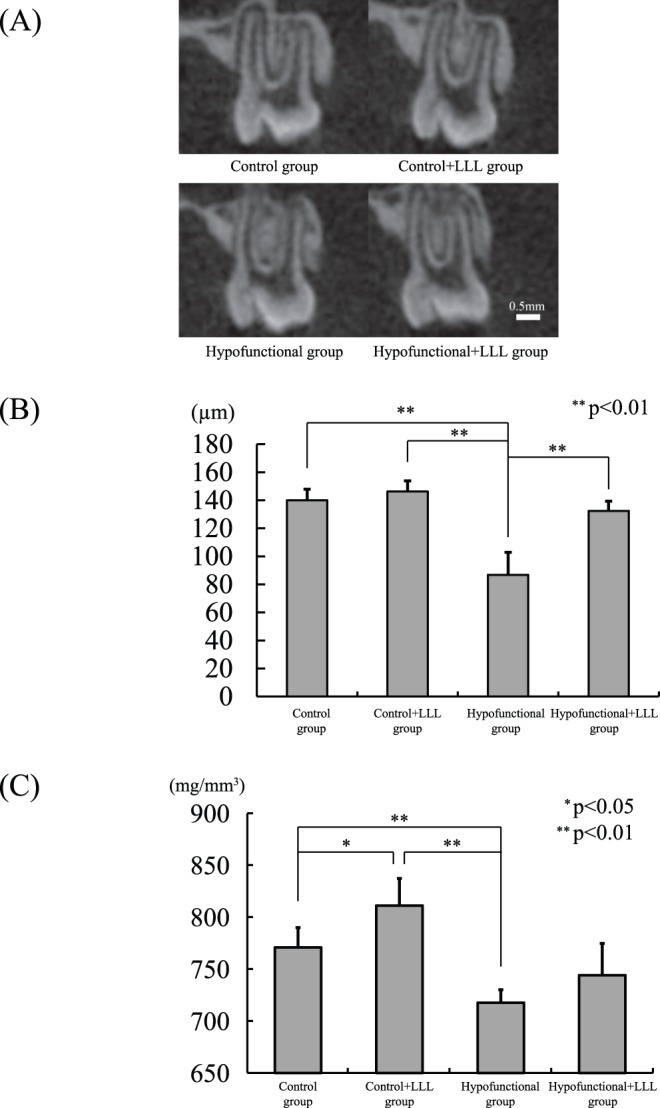
PDL thickness and BMD of in the alveolar bone. (A) Micro-CT images of periodontal tissue at 7 weeks of age. (B) Comparison of PDL thicknesses between the four groups. (C) Comparison of BMDs between the four groups.

No significant difference was observed in PDL thickness between the four groups at 5 weeks of age (data are not shown). However, the PDL in the experimental groups exhibited morphological changes at 7 weeks of age (Fig. 3A). No significant difference was observed in PDL thickness between the control, hypofunctional+LLL, and control+LLL groups at 7 weeks of age. In contrast, PDL thickness was significantly smaller in the hypofunctional group than in the other groups (Fig. 3B).

### BMD in the Maxillary Alveolar Bone

No significant differences were observed in BMD in the maxillary alveolar bone between the four groups at 5 weeks of age (data not shown). BMD in the maxillary alveolar bone for the four groups is shown in Figure 3C. BMD was significantly lower in the hypofunctional group than in the controls. On the other hand, BMD was higher in the hypofunctional+LLL group than in the hypofunctional group, and No significant difference was observed between the control and hypofunctional+LLL groups.

### Histomorphometric Findings

**Figure 4:**
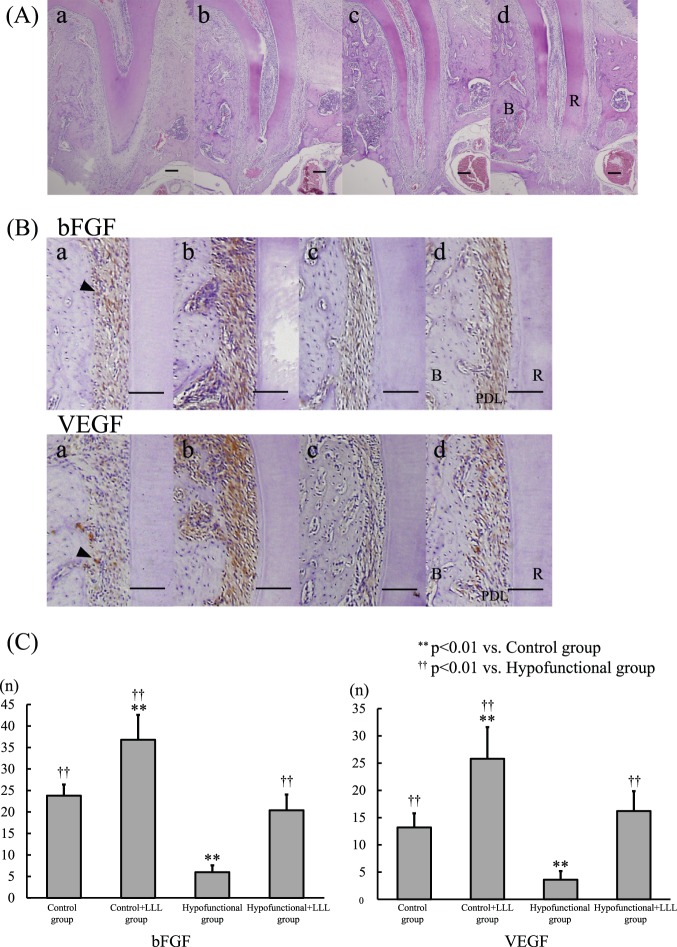
H-E staining and Immunohistochemical staining for bFGF and VEGF. (A) H-E staining. H-E stained sections of rat periodontal tissue. (a) control group, (b) control+LLL group, (c) hypofunctional group, (d) hypofunctional+LLL group. R: root, B: alveolar bone, Bar = 100 µm. (B) Immunohistochemical staining for bFGF and VEGF. One of the bFGF- and VEGF-positive cells in the control group (arrow head) was selected to determine the density for cell counting. (a) control group, (b) control+LLL group, (c) hypofunctional group, (d) hypofunctional+LLL group. R: root, B: alveolar bone, PDL: periodontal ligament, Bar = 100 µm. (C) Comparison of the number of bFGF- and VEGF-positive cells between the four groups.

Figure 4A shows H-E-stained distal palatal root sections from the four groups. In the hypofunctional group, changes in stricture on the PDL were observed, and PDL thickness was thinner than in the other groups. In addition, PDL thickness in the hypofunctional+LLL group was similar to that in the control and control+LLL groups.

### Immunohistochemical Findings of bFGF and VEGF Expression

The number of bFGF and VEGF immunopositive cells, particularly fibroblastic cells, cementum, and alveolar bone lining cells was significantly lower in the hypofunctional group than in the other groups (*P*<0.01; Fig. 4B, 4C).

However, the number of bFGF and VEGF immunopositive cells was significantly higher in the hypofunctional+LLL group than in the hypofunctional group, and was similar to that in the control group (*P*<0.01; Fig. 4B, 4C).

## Discussion

The present study was designed to investigate the influence of LLL on hypofunctional teeth. We developed an experimental hypofunctional model in the molar region using a bite-raising appliance [4]. This method made it possible to simulate hypofunctional conditions in the molar region.

In the present study, occlusal hypofunction using a bite-raising appliance resulted in changes in the periodontal tissue; PDL thickness and BMD in the maxillary alveolar bone were thinner and lower, respectively, than these in the controls. Numerous studies have reported structural changes in the PDL [1], [2] and loss of BMD in alveolar bone [5] with occlusal hypofunction. However, PDL thickness and BMD in the maxillary alveolar bone were thicker and higher, respectively, in the hypofunctional+LLL group than in the hypofunctional group, and no significant difference was observed from the control group. Occlusal stimuli have been shown to affect periodontal tissue; root size and the structure of the PDL may be reduced because of disuse atrophy resulting from defects in occlusal function, and atrophy may recover after occlusal stimuli are regained [4]. The effects of occlusal stimuli on alveolar bone formation have also been reported previously [5]. On the other hand, LLL may increase BMD [11]. Due to its wavelength characteristics, low-level diode lasers are able to reach periodontal tissues, and laser irradiation is expected to induce the proliferation of osteoblasts [14] and periodontal ligament fibroblasts [15]. Therefore, LLL affects periodontal tissue, leading to extensive changes in the PDL and increases in BMD in alveolar bone with occlusal hypofunction.

The number of bFGF- and VEGF-immunopositive cells was significantly lower in the hypofunctional group, particularly for fibroblastic cells, cementum, and alveolar bone lining cells, than in the other groups. However, when LLL was applied, the number of bFGF- and VEGF-immunopositive cells was significantly higher than that in the hypofunctional group, and levels were similar to those in the control group. Recent *in vitro* studies indicated that LLL irradiation was able to stimulate the release of growth factors, such as VEGF [17] and bFGF [18]. LLL has also been shown to have a positive effect on bone formation, and the vasculature [6], [7]. On the other hand, occlusal stimuli regulate bFGF in the rat PDL [16]. PDL homeostasis is a complex mechanism involving inflammation, neovascularization, neurogenesis, bone formation and matrix remodeling. bFGF is a potent angiogenic factor, and angiogenesis may be involved in the periodontal regeneration promoted by bFGF. VEGF also increases vascular permeability [22] and is involved in bone remodeling [23], [24]. Constitutive VEGF expression may contribute to periodontal tissue homeostasis by regulating local blood circulation and bone metabolism. Thus, bFGF and VEGF are essential for periodontal remodeling and vascular permeability in PDL. Therefore, the expression of bFGF and VEGF after LLL stimulation may in increase vascular permeability in the PDL of hypofunctional teeth with a reduction in blood vessels.

Malocclusions, such as an open bite, are often encountered in orthodontic practice and that include hypofunctional teeth in orthodontic practice. Root resorption was shown to be more prominent in hypofunctional teeth than in normal teeth during orthodontic tooth movement [3], [26]. Therefore, it is important for hypofunctional teeth with atrophied periodontal tissues to recover their physiological structure and function. The results of the present study suggests that the recovery of periodontal tissue in hypofunctional teeth is possible with LLL prior to orthodontic tooth movement, and this may reduce or prevent root resorption.

In conclusion, occlusal hypofunction during the growth period may weaken periodontal tissue, leading to PDL stricture and decreased BMD in the alveolar bone crest; however, LLL irradiation to hypofunctional teeth led to a periodontal condition similar to that in normal teeth. bFGF- and VEGF-positive fibroblasts and odontoclasts were also observed in the PDL in the hypofunctional+LLL group. Thus, periodontal tissues, in terms of bFGF and VEGF, were enhanced by LLL, which stimulated the structure and function of periodontal tissues.
